# Recovery benefits of using a heat and moisture exchange mask during sprint exercise in cold temperatures

**DOI:** 10.1177/2050312117740985

**Published:** 2017-11-28

**Authors:** John G Seifert, Jeremy Frost, John A St Cyr

**Affiliations:** 1 Movement Sciences Laboratory, Montana State University, Bozeman, MT, USA; 2 St. Cloud State University, St. Cloud, MN, USA; 3 Jacqmar, Inc., Minneapolis, MN, USA

**Keywords:** Vasoconstriction, blood pressure, cold exposure, performance, thermal mask

## Abstract

**Objectives:**

Breathing cold air can lead to bronchoconstriction and peripheral vasoconstriction, both of which could impact muscular performance by affecting metabolic demands during exercise. Successful solutions dealing with these physiological changes during exercise in the cold has been lacking; therefore, we investigated the influence of a heat and moisture exchange mask during exercise in the cold.

**Methods:**

There were three trial arms within this study: wearing the heat and moisture exchange mask during the rest periods in the cold, no-mask application during the rest periods in the cold, and a trial at room temperature (22°C). Eight subjects cycled in four 35 kJ sprint sessions with each session separated by 20 min rest period. Workload was 4% of body mass.

**Results:**

Mean sprint times were faster with heat and moisture exchange mask and room temperature trial than cold, no-mask trial (133.8 ± 8.6, 134.9 ± 8.8, and 138.0 ± 8.4 s (p = 0.001)). Systolic blood pressure and mean arterial pressure were greater during the cold trial with no mask (15% and 13%, respectively), and heart rate was 10 bpm less during the third rest or recovery period during cold, no mask compared to the heat and moisture exchange mask and room temperature trials. Subjects demonstrated significant decreases in vital capacity and peak expiratory flow rate during the cold with no mask applied during the rest periods.

**Conclusions:**

These negative responses to cold exposure were alleviated by the use of a heat and moisture exchange mask worn during the rest intervals by minimizing cold-induced temperature stress on the respiratory system with subsequent maintenance of cardiovascular function.

## Introduction

Numerous outdoor activities take place during the winter months in cold climates. Some of these activities are aerobic in nature, while others are of high intensity and short duration, such as cross-country skiing sprint races. Cross-country ski races involve intense, physiological stress, taxing the pulmonary, cardiovascular, and skeletal muscular systems.

Peripheral vasoconstriction, influence by thermoreceptors in the skin, and bronchoconstriction play important roles in athletes’ physical performance during cold exposure by changing peripheral blood flow patterns and pulmonary gas exchange. In fact, numerous authors have reported that both anaerobic and aerobic performance is significantly reduced during cold exposure.^1–4^ Patton and Vogel^4^ noted a 38% decrease in the time to exhaustive exercise at 78% maximal oxygen uptake during −20°C cold exposure. Hackney et al.^5^ reported a 5.5% decrease in mean power and a 4.6% decrease in peak power output after cold exposure.

Cardiac ischemia, peripheral vasoconstriction, and bronchoconstriction increase as the pulmonary system is exposed to cold/dry air.^6,7^ The bronchial airway provides the necessary heat and water to warm and humidify the cold, dry inspired air.^8^ Airway cooling leads to vascular reactivity when inspiration of cold air exceeds warming capacity.^9,10^ Some authors report that airway heat loss results in bronchoconstriction,^6,11,12^ while others note that evaporative water loss and hyperosmolality of the mucosal cells lead to bronchoconstriction.^8,13^ Regardless of the mechanisms, the results of cold air inhalation can cause bronchoconstriction and coupled with cutaneous thermoreceptors can influence vascular tissue bed reactivity.

Therefore, might these cold-induced decrements in performance and physiological function be mitigated by an intervention that minimizes cold air inhalation? The use of a heat and moisture exchange (HME) mask, worn over the mouth and nose, aids in warming inspired cold air. The airway chamber of the mask contains a thermal medium of copper that acts as a heat sink by trapping expired heat and water vapor. Heat is released via latent transfer upon cold air inhalation. Thus, the inspired cold air is warmed and humidified as it passes through the chamber in the mask. Warmed, humidified air reduces cardiovascular and respiratory stresses compared to cold air alone.^7,14,15,16^

With the high ventilations observed during high-intensity work, it is plausible that cold air inhalation could negatively affect performance. It is not known, however, if an HME will warm and humidify the cold air sufficiently to have an influence on high-intensity exercise performance. Thus, this study investigated the influence of a HME on cardiopulmonary function during repeated sprint performance exercise in the cold and further to compare sprint performance in the cold to room temperature (RT). We propose that the use of an HME may result in an improved performance and lower cardiopulmonary stress compared to no mask. Furthermore, exercising in a cold condition could affect performance when compared to RT exercise.

## Materials and methods

### Subjects

The intention of this prospective, non-randomized study was to investigate the recovery benefits of performance and hemodynamic and pulmonary function parameters of using a HME mask during sprint exercise in a cold environment. Based on data from previous studies, mean and standard deviations were used with a power analysis of the chosen endpoints in this study to establish that eight subjects were required for this study. Following written approval from the Institutional Review Board prior to enrolling subjects, eight healthy (two females and six males) subjects volunteered to participate. This study commenced and was completed during February and March. Written informed consent was provided by the subjects prior to data collection. Subjects ranged in age from 21 to 28 years (mean 23.4 years) and were accustomed to high-intensity exercise. The average height of the subjects was 1.79 ± 0.07 m, average weight was 75.9 ± 8.4 kg, and their average body mass index (BMI) was 23.9 ± 3.9. Three subjects were competitive cross-country skiers, two were competitive cyclists, and three were well-trained, but non-competitive, exercisers.

Subjects refrained from exercise, alcohol, and caffeinated beverages for 24 h before their trials. Subjects were instructed to maintain similar dietary habits and to refrain from exercise for 24 h prior to the trials. Each subject was instructed to eat and drink 3 h prior to the scheduled testing. All exercise trials were conducted 3–5 h post prandial, but were consistent within each subject. Layers of clothing were similar for both cold trial arms. The insulated clothing was only worn during the rest periods, which consisted of a base layer, sweatshirt, ski jacket, mittens, ski pants, and a ski hat at rest. For each exercise session, the subjects wore the base layer, sweatshirt, mittens, ski hat, and tights on their legs. Subjects removed their jackets for the exercise. Subjects were dressed in shorts and t-shirt for the RT trial.

### Procedures and equipment

Exercise consisted of cycling three 35-kJ test sprint sessions and one practice trial, all of which were performed with a resistance of 4% of body weight on a Monark #868 ergometer (mean resistance: 3.1 ± 0.1 kg). Following collection of baseline data (blood pressure (BP), heart rate (HR), mean arterial pressure (MAP), vital capacity (VC), inspiratory capacity (IC), expiratory reserve volume (ERV), forced expiratory volume (FEV1), and peak expiratory flow rate (PEFR)), all subjects were given a 10 min warm up at 100 W immediately before the first sprint session, and in addition, a 2 min warm up was employed before each subsequent sprint trial period. Each sprint session was separated by a 20 min seated rest period, as determined from previous studies.

**Figure 1.**
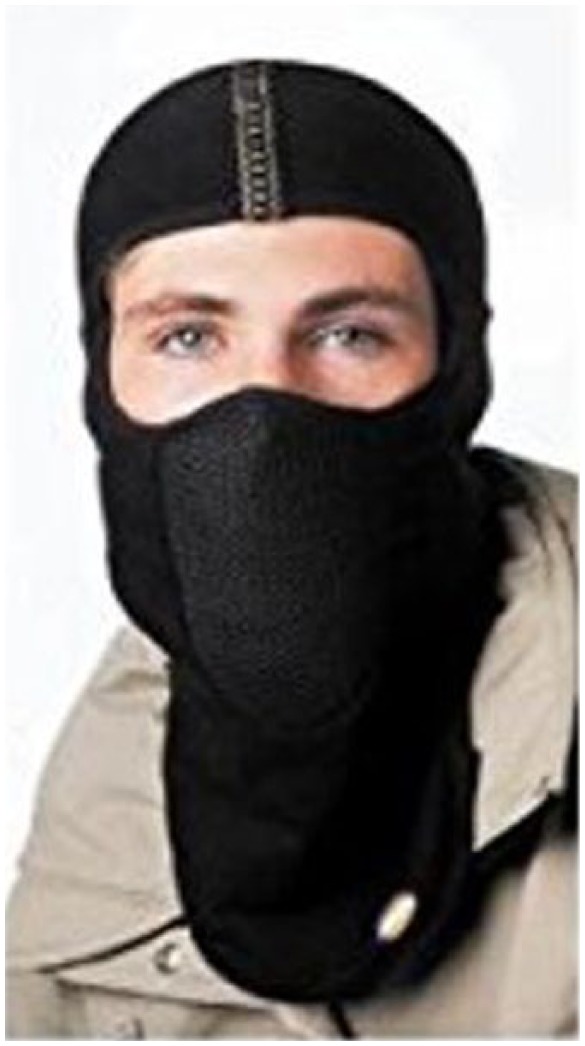
The PolarWrap full head cover exchanger mask was designed, engineered, and manufactured to keep an individual “toasty” by warming the air you breathe by the built-in module of copper coils and mesh that captures the exhaled heat and water vapor on the copper coils and through latent heat transfer warms and humidifies the cold dry air (PolarWrap website, 2017).

As previously stated, subjects completed one practice sprint session at RT and three experimental sprint trials. The practice trial was used to minimize the learning effect. The three trials were counterbalanced and each trial was separated by at least 1 week. One experimental trial was performed at an RT of 22°C, while two trials were completed at −9.0°C ± 0.3°C. To minimize variables, humidity during all trials was kept constant. All trials were performed in windless conditions. Furthermore, the volume of the room was 3 m × 5 m × 3 m and the air was constantly circulating and exchanged to maintain CO_2_ levels at environmental levels, around 0.03%. The temperature variation was ±2° C. During one cold trial, subjects wore an HME, covering the face and neck areas during the warm-up and rest periods (PolarWrap, Inc., Memphis, TN, USA; Figure 1). On the other cold trial, subjects did not wear the mask (cold with no mask (CNM)). Subjects remained in the cold chamber during the rest periods for both of the HME and CNM trials.

Pulmonary function testing (Med Graphics, St. Paul, MN) was completed at RT, before subjects entered the cold and 10 minutes following the fourth sprint. Each subject underwent pre- and post-exercise evaluation of pulmonary functions for all test groups using Spirometry, HR (Polar HR, Stamford, CT) and BP following 10 min of seated rest at RT and at the 16-min mark of each 20-min rest period.

### Statistical analysis

Data were statistically analyzed by an analysis of variance (ANOVA) with repeated measures. Tukey’s post hoc test was used to differentiate means. Data were presented as mean ± standard deviation. Alpha level of p < 0.05 was used for statistical significance.

## Results

All subjects completed the experimental exercise trials without incident. Time to complete the three sprints were significantly faster during the HME trial than the CNM trial (132.9 ± 8.1 s and 131.3 ± 7.7 s vs 139.8 ± 8.5 s and 138.6 ± 8.2 sec, respectively, a 6% improvement in performance (p = 0.008 and 0.01)). No differences, however, were observed for sprint finishing times between HME and RT. The average time to complete the four sprints for HME (133.8 ± 8.6 s) and RT (134.9 ± 8.8 s) trials were significantly faster compared to 138.0 ± 8.4 s for the CNM condition (p = 0.001).

Hemodynamically, resting HR was significantly greater during the HME and RT trials than during the CNM trial (p < 0.000, Table 1). During the cold exposure, resting HR during the rest periods were about 10% lower in the CNM trial compared to HME and RT trials (Table 1). No difference was observed for HR between the HME and RT trials.

**Table 1.** Cardiovascular responses.

Treatment	Rest interval	Heart rate^a^	Systolic BP^a^	Diastolic BP^a^
RT	Pre-exercise	64.5 (3.3)^b^	122.7 (2.5)	72.7 (2.9)
	1	89.1 (4.8)	123.1 (2.3)	74.0 (3.6)
	2	101.6 (4.9)	122.2 (2.8)	77.8 (2.8)
	3	98.9 (5.56)	118.1 (2.0)	74.5 (2.9)
HME	Pre-exercise	64.4 (3.8)^b^	123.7 (2.5)	76.6 (2.8)
	1	94.0 (3.9)	123.5 (2.4)	76.4 (1.6)
	2	98.1 (3.6)	121.3 (2.1)	76.9 (2.4)
	3	97.9 (3.5)	124.9 (1.9)	76.0 (2.3)
CNM	Pre-exercise	69.0 (2.6)^b^	115.0 (2.0)^b^	74.1 (2.4)^c^
	1	85.4 (4.8)	124.1 (2.9)	76.1 (2.0)
	2	89.6 (5.3)	130.0 (2.1)	81.1 (1.7)
	3	87.4 (3.6)	132.3 (2.2)	82.0 (1.8)

RT: room temperature; HME: heat exchange mask; CNM: cold with no mask.

Heart rate: bpm; systolic and diastolic blood pressure: mmHg; mean (±SEM).

a Treatment averages at rest intervals for RT and HME are significantly different from CNM.

b Significantly different from rest interval values.

c Significantly different from intervals 2 and 3.

Between-trial analyses indicated that systolic BP and MAP levels were significantly lower during the second and third rest periods for the HME and RT trials compared to the CNM trial (Table 1). The within-trial analysis indicated that systolic BP and MAP values during the rest periods were not different from baseline for either the HME or RT trials. However, mean systolic BP and MAP values increased significantly (15% and 13%, respectively, p = 0.001 and p < 0.001) during the CNM trial. In fact, MAP values at the second and third rest periods were significantly elevated over the baseline value during the CNM trial, but not in HME or RT trials. No differences between trials were observed for diastolic BP (Table 1) and FEV_1_ (Table 2).

**Table 2.** Pulmonary function.

Treatment	Time	VC	IC	ERV	FEV1	PEFR
RT	Pre	4.99 (0.31)	3.15 (0.27)	1.85 (0.23)	4.39 (0.28)	10.1 (0.60)
	Post	5.09 (0.33)	3.22 (0.21)	1.88 (0.16)	4.46 (0.31)	9.9 (0.69)^a^
HME	Pre	5.10 (0.29)^b^	3.39 (0.20)	1.70 (0.13)	4.48 (0.29)	10.3 (0.76)^b^
	Post	5.29 (0.34)^c^	3.51 (0.23)	1.78 (0.17)	4.54 (0.31)	10.9 (0.78)^c^
CNM	Pre	5.30 (0.29)^b^	3.37 (0.30)	1.94 (0.19)	4.55 (0.28)	10.7 (0.86)^b^
	Post	5.10 (0.30)	3.29 (0.23)	1.81 (0.12)	4.47 (0.28)	10.3 (0.88)

VC: vital capacity (L); IC: inspiratory capacity (L); ERV: expiratory reserve volume (L); FEV1: forced expiratory volume (L); PEFR: peak expiratory flow rate (L/s); RT: room temperature trial; HME: heat exchange mask; CNM: no mask in the cold.

Mean (+SEM).

a Significantly different from HME post-exercise value.

b Significantly different from post-exercise value.

c Post-exercise value is significantly different from CNM post-exercise value.

Subjects on the HME trial experienced significant increases in VC and PEFR from pre-test to post-test exercise, respectively (p = 0.02 and p = 0.01, Table 2). During the CNM trial, however, subjects experienced significant decreases in VC and PEFR (p = 0.000 and p = 0.05, respectively, Table 2).

## Discussion

This study assessed the influence of HME on cardiopulmonary function during repeat sprint performance exercise in a cold environment with a further comparison of a cold condition to RT. Sprint performance in the cold was enhanced when an HME was worn compared to no mask. The HME also produced benefits in measured cardiovascular hemodynamic parameters, as well as an improvement in some measured parameters of pulmonary function while exposed to cold air compared to the CNM trial. The performance and physiological responses in the cold during the HME trial were similar to those from the RT trials.

Hackney et al.^5^ reported that anaerobic sprint performance decreased significantly during cold exposure performed before and after 4.5 days of military field operations in a temperature range from −2°C to −22°C. These authors noted that unavailable substrates, buffering insufficiency, muscle tissue damage, and dehydration may have led to the decrement in anaerobic sprinting performance, not necessarily limited to exercise in the cold. There may be an alternative explanation from those put forth by Hackney et al., which could account for the performance decrement observed only in the CNM trial and not the HME arm of this study. It is unlikely that subjects in this study suffered from the same acute effects as noted by Hackney et al. since they completed the exercise protocol, on average, 4 h post prandial, ingested 200 mL of water 60 min prior to exposure, and did not exercise for 24 h before their trials. Similarly, Faulkner et al.^17^ and Ferretti et al.^1^ noted detrimental changes in muscle performance with decreases in temperature during exercise. Faulkner et al.^17^ observed a decrease in force production as muscle temperature decreased, while Ferretti et al.^1^ reported that anaerobic power has a direct relationship with temperature since adenosine triphosphate (ATP) hydrolysis was reduced when muscle temperature decreased by 8°C. Although not measured in this study, it is doubtful that the cyclists’ muscle temperatures decreased to the extent noted by Ferretti et al. because of the short duration of the rest periods between the sprints and subjects who wore insulated clothing during the rest period. Since there were no statistical differences in performance and physiological variables between HME and RT trials, the most plausible explanation for enhancing performance is to minimize physiological stress by warming the cold inspired air during the rest or recovery periods. Theoretically, the results of this study suggest that the inhalation of cold air influenced sprint performance by effecting changes in vascular and bronchial diameters due to temperature-generated reactivity.

Kippelen and Anderson^14^ state that maintenance of airway integrity is very important due to its role as a physical barrier against the environmental toxins and injuries. These epithelial cells also play a role in modulating inflammation and our immune response.^14^ Certain conditions or states can injure our airway epithelium, such as high-level exercise or when one exercises in cold dry air or in polluted air. The subsequent injury-repair scenario can contribute to the development of bronchial hyper-responsiveness, which has been observed in elite athletes.

Recovery from exercise in the cold can produce a vasoconstrictive effect in the peripheral vasculature when blood flow is shunted from the periphery to the core, which ultimately can influence peripheral perfusion. Ferretti et al.^1^ reported blood flow, and perfusion of the peripheral tissue beds are reduced as temperature decreased during cold immersion. If blood perfusion is reduced, oxygen supply diminishes with metabolic byproduct accumulation. Presumably, it was airway cooling that significantly changed physiological function during the CNM trial in this study. All subjects wore appropriate attire during both cold trials, so peripheral cooling from skin exposure was minimal compared to the airway cooling. Cold air inhalation and cooling of the body can induce a state of vascular tissue reactivity and bronchoconstriction.^6,9,10^

The mechanisms responsible for cold-induced vascular hyperreactivity and bronchoconstriction are multifocal. Two hormones appear to be strongly related to this constriction. Cruden et al.^18^ reported that cold exposure, and not exercise, can lead to a significant increase in endothelin-1 (ET-1) levels. Airway cooling produces and releases ET-1.^19^ Endothelial cells of the pulmonary vessels and heart produce ET-1 levels during cold exposure, increased central venous pressure, and altitude.^18^ Riska and Seifert (unpublished observation) observed that cold air inhalation led to a significant increase in ET-1 concentration. ET-1 increased by 24% from baseline when subjects were exposed to −16°C temperature, but ET-1 concentrations were maintained at baseline levels when subjects wore an HME. Additionally, significant correlations of ET-1 with BP measurements (positive) and HR (negative) were also observed when subjects did not wear an HME.

Cold exposure may also activate the production and release of norepinephrine.^2,20,21^ Norepinephrine stimulates the adrenergic receptors of smooth muscle, which results in localized peripheral vasoconstriction. However, Watt et al.^22^ reported that ET-1 is a significantly more potent vasoconstrictor than norepinephrine. Alleviating the constrictive responses of ET-1 and norepinephrine and minimizing heat loss appear to be critical components in maintaining physiological function during cold exposure.^2,16,23^ Vasoconstriction can alter BP, HR, and blood flow, establishing a physiological environment that can impair functional performance and muscle recovery.

We found that cardiovascular function was maintained at similar levels during the rest periods for the HME and RT trials. However, systolic BP and MAP increased by 15% and 13% from baseline and HR was 10 bpm lower during the HME and RT trials compared to the CNM trial. This decrease in HR may indicate the presence of a greater circulating central blood volume, as has been reported by previous authors.^24,25^ As central blood volume increases, stroke volume can increase with a decrease in HR.^20,25,26^ BPs were maintained during the CNM trial in this study until approximately 38 min of cold exposure. The increase in BP and decrease in HR observed in CNM implies that the induced peripheral vasoconstriction redirected blood from the periphery to the core.

## Conclusion

In conclusion, the HME provides protection from cold air inhalation, reflected in a benefit in both performance and physiological function. Reasons for the improved performance during cold exposure with the HME are multifactorial; however, the maintenance or improvement in both cardiovascular and pulmonary functions can be preserved by minimizing heat loss via respiratory tract, most pronounced during exercise in cold environments.

### Limitations of the study

This design of this study was not able to blind the subjects to the experimental conditions. Detailed recording of the subject’s body temperature during and following each trial was not performed. The PolarWrap mask is manufactured to cover the face and neck areas. Questions may arise if covering the neck area could play any role in this study. However, previous works by Deal et al.^11^ and McFadden et al.^27^ have clearly demonstrated that it is the cold air inhalation that provokes physiological change. Furthermore, the study involved only eight subjects with meaningful statistical analysis of the data; however, in the future, a larger number of subjects should be considered to verify the statistical results found in this study.
